# Serovar-Dependent Gene Regulation and Antimicrobial Tolerance in *Streptococcus suis* Biofilms

**DOI:** 10.3390/antibiotics14121224

**Published:** 2025-12-04

**Authors:** Mario Delgado-García, Carmen Arenas-Fernández, Oscar Mencía-Ares, Lucía Manzanares-Vigo, Ana Isabel Pastor-Calonge, Alba González-Fernández, César B. Gutiérrez-Martín, Sonia Martínez-Martínez

**Affiliations:** 1 Department of Animal Health, Faculty of Veterinary, Universidad de León, 24071 León, Spain; mdelgg05@estudiantes.unileon.es (M.D.-G.); carenf00@estudiantes.unileon.es (C.A.-F.); lmanv@unileon.es (L.M.-V.); apasc@unileon.es (A.I.P.-C.); algonf@unileon.es (A.G.-F.); cbgutm@unileon.es (C.B.G.-M.); smarm@unileon.es (S.M.-M.); 2 Instituto de Desarrollo Ganadero y Sanidad Animal (INDEGSAL), Universidad de León, 24071 León, Spain

**Keywords:** antibiotic, biofilm, gene expression, serovar, resistance, RNA, RT-qPCR, *Streptococcus suis*

## Abstract

**Introduction:** *Streptococcus suis* is a zoonotic pathogen of great relevance to the swine industry, characterized by high genetic diversity and multiple serovars (SVs) with varying clinical prevalence. Biofilm formation represents a key factor in its virulence, antimicrobial resistance and infection persistence. **Methods:** We integrated gene expression profiling of biofilm-associated genes by RT-qPCR and antimicrobial susceptibility in planktonic and mature biofilm against five antibiotics in *S. suis* field isolates belonging to SV1, SV2, SV7 and SV9. **Results:** Expression of quorum sensing and adhesion genes (*luxS*, *fbps*, *sadP* and *srtA*) was significantly higher in SV2, the poorest biofilm formers, and inversely correlated with biofilm biomass, suggesting these factors act during early biofilm establishment. Correlation analysis indicated coordinated regulation among genes involved in quorum sensing, adhesion and capsule synthesis. Antimicrobial susceptibility testing revealed a high frequency of non-wild type phenotypes in planktonic cells for tetracycline, erythromycin and clindamycin (>80%), while ampicillin and ciprofloxacin were less frequent. Mature biofilms exhibited a significant increase in antimicrobial tolerance for all antibiotics tested, with SV2 showing the greatest susceptibility. **Conclusions:** These data highlight serovar-specific biofilm regulation patterns and enhanced drug tolerance in established *S. suis* biofilms.

## 1. Introduction

*Streptococcus suis* is a Gram-positive bacterium widely known as an important swine pathogen and a cause of zoonotic infections [1]. Despite its pathogenic potential, it is often found as a commensal microorganism in healthy animals, mainly colonizing the upper respiratory tract [2], as well as the gastrointestinal and genital tracts [3]. However, under certain conditions, it can cross mucosal barriers and become an invasive pathogen, entering the bloodstream and spreading to various organs [4], causing diseases such as meningitis, arthritis, pneumonia, endocarditis, septicemia and sudden death [5,6].

Based on its capsular antigens, this encapsulated pathogen was originally classified into 35 serovars (SVs), which were later reduced to 29 after several taxonomic reclassifications [7]. The distribution and prevalence of SVs vary significantly by geographic region. For instance, a study that analyzed more than 8000 isolates collected between 2009 and 2022 across 13 European countries identified SV9 as the most prevalent, followed by SV2, SV1 and SV7 [8]. Similarly, a previous study conducted by our research group on Spanish isolates found SV9 to be the most frequent, followed by SV1, SV2, SV3 and SV7 [9]. Beyond these epidemiological patterns, SVs also differ in clinically relevant traits. Notably, SV2 has been frequently associated with severe systemic disease and is often regarded as one of the most virulent SVs [10]. This serovar-dependent diversity underscores the need to consider both SV and associated virulence factors when evaluating *S. suis* infections [11].

Although more than 100 virulence factors have been defined to be potentially involved in the development of *S. suis* infection in pigs [12,13], the only factor identified as essential for its pathogenicity is its polysaccharide capsule [14]. Its synthesis is encoded by a group of genes known as *cps* (capsule polysaccharide synthesis), which are organized into a specific operon that varies among SVs [15]. Other extensively studied factors include muramidase-released protein, the extracellular protein factor, suilysin [11] or the ability to form biofilm. The latter has gained particular importance in recent years and is currently considered one of the key factors in its pathogenicity [16].

Biofilm is an organized form of bacterial life in which cells aggregate and become embedded in a self-produced matrix composed of substances such as polysaccharides, proteins, lipids and extracellular DNA [17]. Biofilm formation in *S. suis* is closely linked to the regulation of genes involved in bacterial communication and adhesion, with the quorum sensing gene *luxS* playing a central role in coordinating virulence and early biofilm establishment [18], while adhesin-encoding genes such as *fbps*, *sadP* and *srtA* mediate attachment to host tissues and extracellular components [19,20,21].

In *S. suis*, biofilm formation not only contributes to virulence but also strongly enhances persistence by reducing the effectiveness of antimicrobial treatments [22]. While antimicrobial resistance typically arises from heritable changes that reduce susceptibility, most commonly through the acquisition of specific resistance determinants [23], biofilm-associated tolerance reflects a transient, phenotypic decrease in antimicrobial efficacy driven by the physiological state of biofilm-embedded cells rather than by genetic mechanisms [24]. This diminished susceptibility results from several complementary factors, including the horizontal transfer of resistance genes [25], the slow growth and low metabolic activity of biofilm cells that limit the action of antibiotics targeting active processes such as protein or DNA synthesis [26] and the increased expression of defense mechanisms like efflux pumps that expel antimicrobials from the bacterial cell [27]. Additionally, the extracellular matrix acts as a physical barrier that limits antibiotic penetration, while the internal microenvironment of the biofilm reduces nutrient and oxygen availability, promoting a metabolically inactive state that further decreases bacterial sensitivity to treatment [11].

In a previous study conducted by our research group [28], in which the in vitro biofilm-forming capacity of *S. suis* clinical isolates belonging to different SVs was analyzed, significant differences were found among the studied SVs, suggesting a possible differential expression of genes related to biofilm formation among the different SVs. Therefore, this study aims to evaluate the differential expression of key biofilm-associated genes and assess the susceptibility of biofilm-forming *S. suis* isolates to a selection of antimicrobials through a serovar-dependent analysis.

## 2. Results

### 2.1. Gene Expression Analysis in a Selection of Streptococcus suis Isolates

#### 2.1.1. Comparative Analysis of Biofilm-Related Gene Expression

The analysis of biofilm-related gene expression in a selection of 60 *S. suis* isolates by RT-qPCR representing the four most prevalent SVs (SV1, SV2, SV7 and SV9) revealed differences in the transcriptional activity of the selected genes involved in quorum sensing (i.e., *luxS*), adhesion (i.e., *fbps*, *sadP* and *srtA*) and capsule biosynthesis (i.e., *cpsE*) under biofilm-forming conditions.

**Figure 1:**
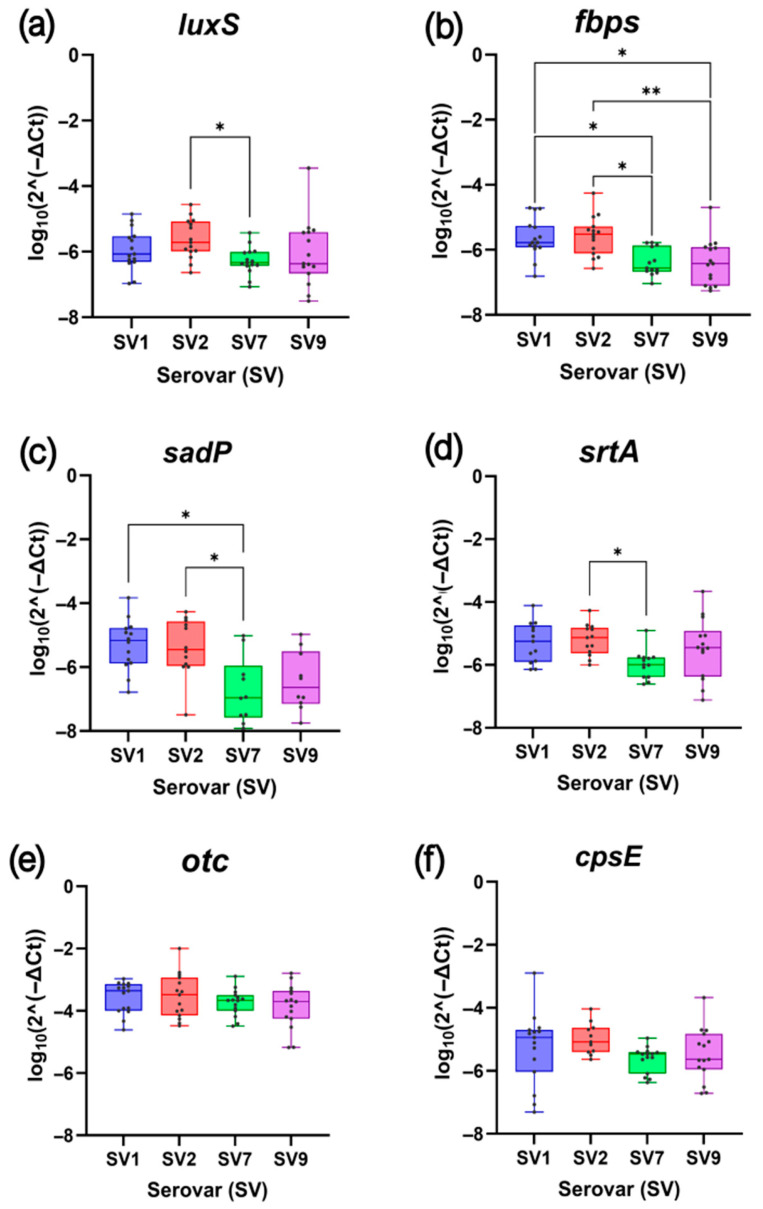
Expression levels of the biofilm-related genes according to the selected *Streptococcus suis* serovars (SVs). (**a**) *luxS*; (**b**) *fbps*; (**c**) *sadP*; (**d**) *srtA*; (**e**) *otc*; (**f**) *cpsE*. The boxplots represent the quartiles and median of the data, and each point corresponds to an individual isolate. Significant differences between serovars are shown with asterisks, including *p* < 0.05 (*) and *p* < 0.01 (**).

**Figure 2:**
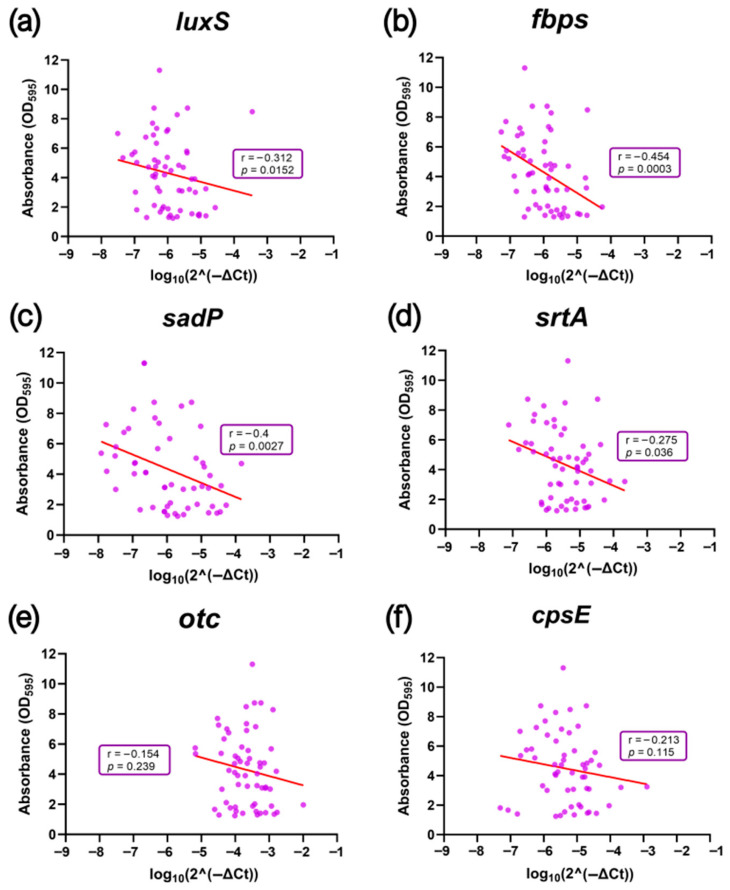
Scatter plots of the relationship between the expression of biofilm-related genes and biofilm formation measured by absorbance (OD_595_) after crystal violet staining in a selection of *Streptococcus suis* isolates. (**a**) *luxS*; (**b**) *fbps*; (**c**) *sadP*; (**d**) *srtA*; (**e**) *otc*; (**f**) *cpsE*. The trend line is included along with the Spearman correlation coefficient (r) and its corresponding *p*-value.

For *luxS*, which participates in quorum sensing through AI-2 synthesis, *S. suis* SV2 isolates displayed the highest expression levels, significantly exceeding those of SV7 (*p* < 0.05) (Figure 1a), which represent the lowest and highest biofilm formers, respectively. Spearman correlation analysis between gene expression and biofilm formation using the crystal violet (CV) method confirmed a moderate but significant negative association between *luxS* expression and biofilm formation capacity (r = −0.312; *p* < 0.05) (Figure 2a). This indicates that isolates with higher *luxS* expression tend to be less efficient at forming biofilms.

Regarding the *fbps*, *sadP* and *srtA* genes, each involved in *S. suis* adhesion to host cells, a similar expression pattern was observed (Figure 1b–d), highlighting significantly higher expression levels (*p* < 0.05) in SVs with lower biofilm-forming capacity, such as SV2, compared to those with higher levels, especially SV7. Spearman correlation analysis (Figure 2b–d) also revealed a statistically significant moderate negative correlation between gene expression and biofilm formation in all three cases, particularly for *fbps* (r = −0.454, *p* < 0.001). These results suggest that isolates with lower biofilm-forming ability tend to express adhesion-related genes at higher levels.

For the *otc* gene, which is involved in the arginine deiminase system, no significant differences in expression levels were found between the *S. suis* SVs studied (Figure 1e). Its expression was consistently high across isolates, and the Spearman correlation analysis revealed a weak, non-significant negative association between *otc* expression and biofilm formation levels (r = −0.154, *p* = 0.239) (Figure 2e).

Finally, expression of the *cpsE* gene, involved in capsule biosynthesis, showed limited variation among serovars (Figure 1f). Although differences did not reach statistical significance, a trend toward higher expression in *S. suis* SV2 compared to SV7 was observed (*p* = 0.06). Correlation analysis revealed a weak negative trend (r = 0.213, *p* = 0.115) (Figure 2f), suggesting that isolates with lower biofilm-forming capacity tend to upregulate capsule biosynthesis genes.

#### 2.1.2. Correlation Analysis of Gene Expression Levels

**Figure 3:**
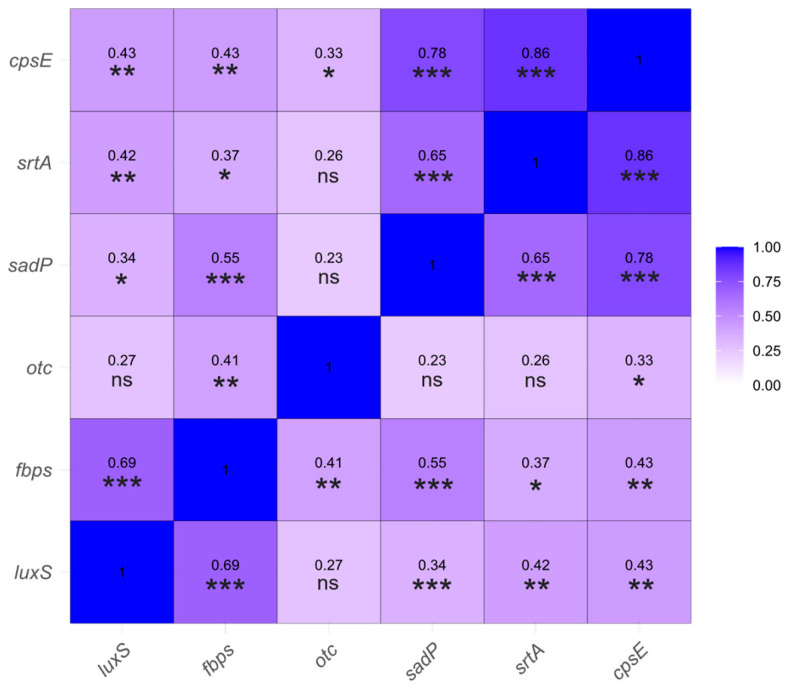
Spearman correlation matrix of relative expression levels (log_10_(2^(−ΔCt)^) of the biofilm-related genes analyzed in this study. Each cell represents the Spearman correlation coefficient (r) between two genes, indicating the strength and direction of their association. Asterisks indicate the significance of the correlation (*p* < 0.05 (*), *p* < 0.01 (**), *p* < 0.001 (***)), while “ns” denotes non-significant results.

To explore potential associations between gene expression levels, a Spearman correlation matrix was generated (Figure 3). This analysis helps to identify possible patterns of functional relationships or shared regulatory mechanisms in those genes involved in capsule synthesis, adhesion and biofilm formation.

The strongest associations were observed between *srtA* and *cpsE* (r = 0.86, *p* < 0.0001), *sadP* and *cpsE* (r = 0.78, *p* < 0.0001), and *sadP* and *srtA* (r = 0.65, *p* < 0.0001). Additional significant correlations included those of *fbps* with *luxS*, *sadP*, *cpsE* and *srtA* (r = 0.69, *p* < 0.0001; r = 0.55, *p* < 0.0001; r = 0.43, *p* < 0.01; r = 0.37, *p* < 0.05, respectively). Likewise, *luxS* showed moderate correlations with *srtA* (r = 0.42, *p* < 0.01), *cpsE* (r = 0.43, *p* < 0.01) and *sadP* (r = 0.34, *p* < 0.05). Finally, *otc* expression correlated significantly with *cpsE* (r = 0.33, *p* < 0.05) and *fbps* (r = 0.41, *p* < 0.01). As no correction for multiple comparisons was applied, weaker associations should be interpreted cautiously; however, the strongest correlations (r ≥ 0.65) are unlikely to be affected by this limitation and can be considered robust.

### 2.2. Antimicrobial Resistance Assays and Biofilm Characterization in Streptococcus suis

#### 2.2.1. Impact of Biofilm Lifestyle on the Wild-Type and Non-Wild-Type Phenotypes of *Streptococcus suis* Against Commonly Used Antibiotics

Here, we evaluated the antimicrobial susceptibility profiles of the 60 *S. suis* isolates selected against five antibiotics (i.e., ampicillin, tetracycline, erythromycin, clindamycin and ciprofloxacin) using three complementary methodologies.

**Figure 4:**
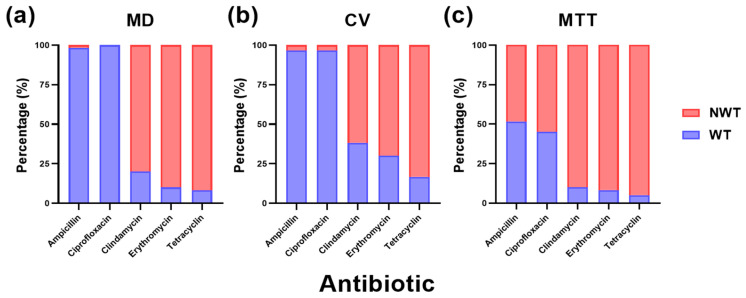
Proportion (as percentage) of wild-type (WT) and non-wild-type (NWT) *Streptococcus suis* isolates per antibiotic determined by three antimicrobial susceptibility testing methods. (**a**) Broth microdilution (MD); (**b**) crystal violet (CV) assay; (**c**) MTT assay. Bars show the percentage of WT (blue) and NWT (red) isolates for the selected antibiotics.

The first methodology intended to determine wild-type (WT)/non-wild-type (NWT) phenotypes under planktonic conditions using the broth microdilution (MD) method. It revealed that the NWT phenotype was predominant for tetracycline (91.7%, *n* = 55), erythromycin (90%, *n* = 54) and clindamycin (80%, *n* = 48). Conversely, the frequency of NWT isolates for ampicillin (1.7%, *n* = 1) was particularly low and no resistance was detected to ciprofloxacin (Figure 4a).

The second methodology consisted of evaluating the capacity of biofilm formation in the presence of antibiotics via staining with CV after removing the broth, in which planktonic bacteria may exist. It was observed that there was a reduction in antimicrobial tolerance for all antibiotics analyzed, except for ampicillin and ciprofloxacin, which maintained a low biofilm-mediated tolerance (3.3%; *n* = 2). The rest of antibiotics showed the following percentage of biofilm-mediated tolerance: 83.3% (*n* = 50) for tetracycline, 70% (*n* = 42) for erythromycin and 61.7% (*n* = 37) for clindamycin (Figure 4b).

Finally, the third method evaluated bacterial survival when antimicrobials were applied to pre-established biofilms, using an MTT-based cell viability assay. The results showed a significant (*p* < 0.05) increase in the proportion of antimicrobial tolerance after treatment under biofilm conditions. While the percentages of biofilm-mediated tolerant for tetracycline (95%, *n* = 57), erythromycin (91.7%, *n* = 55) and clindamycin 90%, *n* = 54) remained relatively similar to those obtained with previous methods, the most pronounced increases were observed for ampicillin and ciprofloxacin. In these cases, the proportion of antimicrobial tolerance increased dramatically, from nearly absent levels in planktonic conditions to 55% (*n* = 33) and 48.3% (*n* = 29), respectively (Figure 4c).

#### 2.2.2. Impact of Antimicrobial Susceptibility Methods on the Minimum Inhibitory Concentration

To further investigate these observations and evaluate if differences in bacterial susceptibility to antibiotics depend on the evaluation method used, minimum inhibitory concentration (MIC) values obtained through the three methodologies were analyzed (Tables S1–S3).

**Figure 5:**
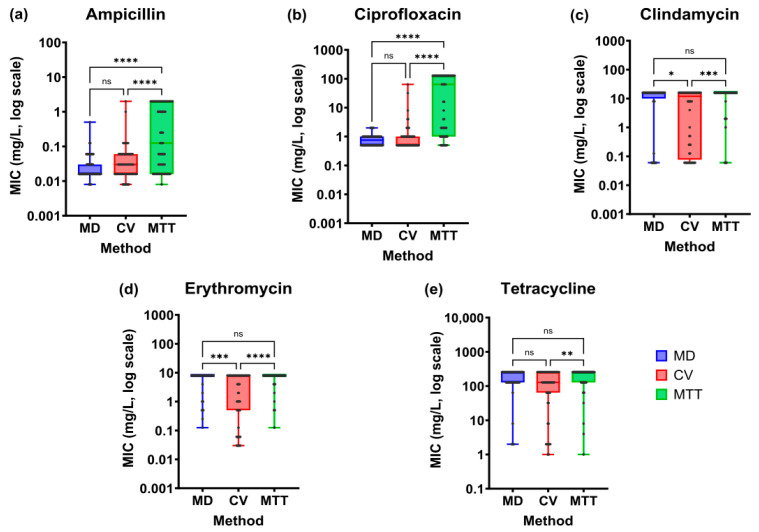
Distribution of minimum inhibitory concentration (MIC) values obtained with each method, i.e., broth microdilution (MD), crystal violet (CV) assay and MTT assay, for the studied antibiotics. (**a**) Ampicillin; (**b**) ciprofloxacin; (**c**) clindamycin; (**d**) erythromycin; (**e**) tetracycline. The boxplots represent the quartiles and median of the data, and each point corresponds to an individual isolate. Significant differences are shown (*p* < 0.05 (*), *p* < 0.01 (**), *p* < 0.001 (***), *p* < 0.0001 (****)), while “ns” denotes non-significant results.

The Friedman test results revealed statistically significant differences in the MIC values among the three methods for all the antibiotics evaluated, ampicillin (*p* < 0.0001), clindamycin (*p* < 0.001), ciprofloxacin (*p* < 0.0001), erythromycin (*p* < 0.001) and tetracycline (*p* < 0.01), demonstrating the impact of the bacterial physiological state on its antimicrobial tolerance. Further pairwise comparisons showed a common pattern for most antibiotics. The MTT method yielded significantly higher MIC values compared to MD and CV techniques, reflecting increased tolerance of cells in biofilm state (Figure 5). For ampicillin (Figure 5a) and ciprofloxacin (Figure 5b), MIC values obtained with MTT were markedly higher, with significant differences relative to both MD and CV (*p* < 0.0001), whereas no differences were found between MD and CV. For clindamycin (Figure 5c), erythromycin (Figure 5d) and tetracycline (Figure 5e), CV staining produced significantly lower values compared to the other methods. However, no differences were found between MD and MTT (*p* > 0.05).

#### 2.2.3. Comparative Analysis of Serovar-Specific Antimicrobial Susceptibility

**Figure 6:**
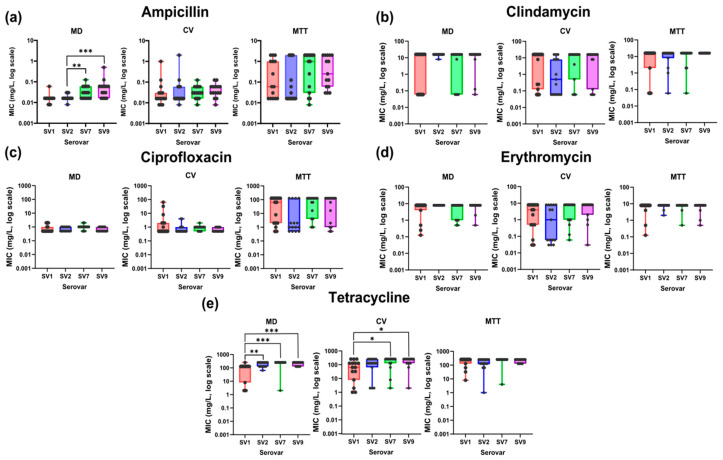
Distribution of minimum inhibitory concentration (MIC) values obtained with each method, i.e., broth microdilution (MD), crystal violet (CV) assay and MTT assay, for the studied antibiotics, stratified by *S. suis* SVs (SV1, SV2, SV7 and SV9). (**a**) Ampicillin; (**b**) clindamycin; (**c**) ciprofloxacin; (**d**) erythromycin; (**e**) tetracycline. The boxplots represent the quartiles and median of the data, and each point corresponds to an individual isolate. Significant differences are shown (*p* < 0.05 (*), *p* < 0.01 (**), *p* < 0.001 (***)).

The analysis of MIC values among *S. suis* SVs revealed significant differences between certain antibiotics and testing methods (Figure 6). The most pronounced variations were observed for ampicillin (Figure 6a) and tetracycline (Figure 6e), while clindamycin, ciprofloxacin and erythromycin showed homogeneous susceptibility profiles (Figure 6b–d).

For ampicillin, the Kruskal–Wallis test indicated significant differences in MD method (*p* < 0.001), but not in CV (*p* > 0.05) or MTT (*p* = 0.05) assays. Pairwise Wilcoxon tests confirmed that SV2 had significantly lower MIC values than SV7 (*p* < 0.01) and SV9 (*p* < 0.001), reflecting greater susceptibility under planktonic conditions. However, these differences disappeared under biofilm conditions.

Tetracycline also showed significant variation in MD (*p* < 0.001) and CV (*p* < 0.05) assays, but not in MTT (*p* > 0.05). SV1 consistently displayed lower MICs than SV2 (*p* < 0.01), SV7 (*p* < 0.001) and SV9 (*p* < 0.001) in MD method and lower values than SV7 (*p* < 0.05) and SV9 (*p* < 0.05) in CV assay, confirming its higher susceptibility, although these differences were not detected in MTT.

## 3. Discussion

Biofilm formation in *S. suis* represents a major challenge as it enhances bacterial persistence, protects against antimicrobial treatment and complicates infection control [16,17,18]. Beyond its role in virulence, biofilm critically influences therapeutic response, highlighting the importance of understanding its genetic and phenotypic determinants. In the present study, we show that quorum sensing and adhesion gene regulation are serovar-dependent, with higher expression in isolates with limited biofilm-forming capacity, indicating that these factors are mainly active during the early stages of colonization. Our findings also revealed that preformed biofilms markedly increased antimicrobial tolerance, particularly reducing tolerance to ciprofloxacin and ampicillin. Altogether, these results indicate that high gene expression predominates in early biofilm development, whereas maturation enhances the protective capacity of the biofilm, leading to increased antimicrobial tolerance.

Understanding how *S. suis* regulates biofilm formation at the genetic level is essential to explain the phenotypic differences observed among serovars. The analysis of gene expression revealed that genes involved in quorum sensing and adhesion (i.e., *luxS*, *fbps*, *sadP* and *srtA*) showed similar expression profiles among *S. suis* serovars. The *luxS* gene is involved in the synthesis of autoinducer-2 (AI-2), a signaling molecule crucial for quorum sensing and bacterial communication, which plays a significant role in regulating biofilm formation [18]. Previous studies have shown that *luxS* mutants exhibit reduced biofilm formation [29], while *luxS* overexpression or the addition of AI-2 enhances this ability [30,31], confirming its key role in regulating early biofilm development. In our study, the significantly higher *luxS* expression observed in *S. suis* SV2, despite its reduced biofilm-forming capacity, could reflect the peak transcriptional activity of the gene during the initial stages of biofilm establishment, when bacterial metabolism and gene expression remain highly active [32]. As the biofilm matures and cells shift toward a more stationary and metabolically inactive state [33], *luxS* expression likely decreases, as we observed in stronger biofilm formers such as SV7.

Similarly, the adhesion-related genes *fbps*, *sadP* and *srtA*, which are essential for initial attachment and colonization, followed the same pattern. *fbps* encodes a fibronectin-binding protein that facilitates adherence to host extracellular matrix components, while *sadP* is a galabiose-dependent adhesin that mediates adhesion to host cells. *srtA* encodes sortase A, an enzyme that anchors adhesins to the bacterial cell wall, supporting the establishment of infection and biofilm formation [19,20,21]. In our study, *S. suis* SV2 exhibited higher transcript levels of these genes compared to SVs capable of forming more structured biofilms, supporting their predominant role during early stages of colonization. Taken together, the coordinated upregulation of *luxS* and adhesion genes in low biofilm formers, particularly SV2, suggests that these factors are predominantly active during the early and metabolically dynamic stages of biofilm development, supporting initial colonization and surface attachment. In contrast, SVs capable of forming mature, structured biofilms, such as SV7, show reduced expression of these genes, reflecting a transition toward a more stable and transcriptionally quiescent state as biofilms mature. However, this interpretation remains inferential, as gene expression was assessed at a single 24-h time point and reflects cross-sectional differences among SVs rather than temporal changes within the same isolate. Future longitudinal, multi-time-point studies will be required to confirm these patterns and further clarify the temporal dynamics of *luxS* and adhesion genes during biofilm development.

In the case of cpsE, which encodes a glycosyltransferase involved in capsule synthesis and includes serovar-specific variants (cps1E, cps2E, cps7E and cps9E), the expression pattern observed tends to align with previous studies showing that during biofilm formation, bacteria reduce capsule production, which promotes adhesin exposure and enhances cell-to-cell cohesion, aiding biofilm establishment [34,35]. Nonetheless, although the correlation was not statistically significant in this study, the observed trend suggests that further studies need to be performed to evaluate its impact in biofilm formation.

Interactions among genes involved in intercellular communication, adhesion and capsule biosynthesis suggest a coordinated regulatory network that underlies key virulence traits in *S. suis*. The strong associations observed between *srtA* and *cpsE* (r = 0.86), and between *sadP* with both *cpsE* (r = 0.78) and *srtA* (r = 0.65), suggested possible co-regulation of genes participating in adhesion and capsule biosynthesis. This aligns with the functional role of sortase A in anchoring capsular components and adhesins to the bacterial cell wall [21] and highlights the interdependence between adhesion mechanisms and capsule production during early colonization [4]. Moreover, the correlations of *fbps* with genes related to quorum sensing, adhesion and capsule formation support the existence of a broader regulatory network in *S. suis*. Likewise, the correlations observed between *luxS* and genes involved in adhesion and capsule synthesis support the role of quorum sensing in regulating virulence factors, consistent with previous studies underscoring the influence of the AI-2 system on *S. suis* pathogenic traits [29]. Finally, the correlations observed between *otc* and genes related to adhesion and capsule synthesis suggest a functional link between metabolic pathways and virulence regulation, which aligns with previous reports describing the integration of metabolic activity and biofilm formation processes [36]. Overall, the correlation analysis suggests coordinated regulation in *S. suis* among genes involved in quorum sensing, adhesion and capsule synthesis, potentially enabling a synergistic response during host colonization and infection. However, further transcriptomic studies are needed to deeply investigate these associations.

Beyond gene regulation, biofilm development also impacts antimicrobial susceptibility, since the structural and physiological changes that accompany biofilm maturation profoundly shape bacterial response to antimicrobials [16,37]. This influence becomes evident when analyzing the WT–NWT phenotype of *S. suis* against commonly used antibiotics. The high NWT rates against tetracycline (91.7%), erythromycin (90%) and clindamycin (80%) observed in planktonic bacteria agrees with previous reports [9,38,39], likely due to their frequent use in swine respiratory infections [40] and to shared resistance mechanisms, particularly between macrolides and lincosamides (i.e., erythromycin and clindamycin, respectively) [33,40]. In contrast, the ciprofloxacin and ampicillin NWT phenotype was particularly low, consistent with previous studies [9,41,42]. These results reflect that the NWT profile of *S. suis* mainly reflects the selective pressure exerted by commonly used antimicrobials in swine production.

When biofilm-associated communities were evaluated, removal of planktonic cells lowered the frequency of NWT isolates, as evaluated with the CV assay, while exposure of mature biofilms to antibiotics markedly increased tolerance. These findings were exacerbated when analyzing MIC values. Interestingly, our results suggest that biofilm formation does not substantially affect susceptibility to ampicillin and ciprofloxacin in early stages, but tolerance sharply increases once biofilms are established, going from 1.7% and absence of NWT isolates to 55% and 48.3%, respectively, which agrees with previous studies [43,44]. This highlights the crucial role of biofilm maturation in enhancing antimicrobial tolerance even to antibiotics generally effective against *S. suis.*

In contrast, clindamycin, erythromycin and tetracycline seem to be affected during the early stages of biofilm development, as evidenced by the significantly lower MIC values obtained with the CV assay compared to the MD and MTT methods, highlighting that antimicrobial response is strongly shaped by the stage of biofilm maturation. As biofilms become established, tolerance appears to be driven by common protective factors such as reduced antibiotic penetration, the presence of the extracellular matrix and persister cells [26]. These findings agree with reports of reduced antimicrobial efficacy in mature biofilms [43,44] and with evidence showing systematically higher MIC values for biofilm-associated bacteria compared to planktonic cells [45]. Altogether, these results indicate that biofilm development substantially reduces antibiotic efficacy, which may lead to persistent infections and therapeutic failures.

Building on the differences observed in antimicrobial response under planktonic and biofilm conditions, our results further revealed that these traits also vary among *S. suis* SVs. Antimicrobial susceptibility in *S. suis* appears to be influenced by serovar-specific characteristics, particularly those related to capsular composition and biofilm-forming capacity [46]. In our study, *S. suis* SV2 exhibited a lower NWT profile and distinct antimicrobial tolerance to ampicillin, consistent with its limited ability to form mature biofilms and supporting the proposed role of the capsule in modulating antimicrobial behavior [41]. These findings suggest that the ability to establish and maintain structured biofilms differs across SVs, directly impacting both persistence and treatment response. Therefore, the interplay between capsule composition and biofilm formation may contribute to the variable antimicrobial behavior of *S. suis*, reinforcing the need to consider serovar-specific features when evaluating therapeutic efficacy.

The antimicrobial tolerance findings highlight the importance of considering biofilm physiology when assessing antimicrobial susceptibility in *S. suis*, as standard methods primarily reflect planktonic behavior [47]. Neglecting biofilm-associated tolerance may lead to underestimated MIC values and therapeutic failures, particularly in chronic infections [47,48]. This is especially relevant for β-lactam antibiotics such as ampicillin, whose reduced activity under biofilm conditions could favor persistence of tolerant strains and treatment inefficacy in vivo [49].

In conclusion, the integration of both studies supports a dynamic model of biofilm formation in *S. suis*. Gene expression analysis revealed that quorum sensing and adhesion genes were more highly expressed in isolates with low biofilm-forming capacity, particularly *S. suis* SV2, reflecting a metabolically active state characteristic of early colonization. In contrast, *S. suis* SVs with stronger biofilm formation, such as SV7 and SV9, showed lower expression of these genes, consistent with a more stationary phase. Complementary to this, the antimicrobial analysis demonstrated that tolerance is greater in mature biofilms, where reduced metabolic activity, the protective matrix and the presence of persister cells confer enhanced survival compared to early stages. Taken together, these results offer an integrated view of gene expression patterns and antimicrobial tolerance in *S. suis*, emphasizing their relevance to understanding *S. suis* persistence and its clinical impact.

## 4. Materials and Methods

### 4.1. Selection of Streptococcus suis Isolates

From the strain collection available from the BACRESPI research group (Animal Health Department, University of León, Spain), a representative set of 60 *S. suis* clinical isolates recovered from Spanish swine farms between 2023 and 2024 was selected. These isolates were evenly distributed (*n* = 15) among the four most relevant serovars (SV1, SV2, SV7 and SV9). The criteria for isolate selection included their previously evaluated biofilm-forming capacity using the crystal violet method [28], along with the prevalence and clinical significance of the SVs in the European context.

These isolates were recovered from cryovials stored at −80 °C in freezing medium and cultured on chocolate agar plates (Oxoid, Basingstoke, UK). Plates were incubated for 24 h at 37 °C under microaerophilic conditions to allow bacterial growth prior to the initiation of experimental procedures.

### 4.2. Gene Expression Analysis by RT-qPCR

#### 4.2.1. Biofilm Formation

After bacterial growth on chocolate agar plates, biofilm formation was initiated. A single cell-isolated colony from each culture was selected and inoculated into one well of a 96-well polystyrene microfibre cell culture-treated plates (Corning Incorporated, Corning, NY, USA) with 200 μL of Todd–Hewitt broth (THB) (Condalab, Madrid, Spain) supplemented with 5% fetal bovine serum (FBS) (Gibco, Norristown, PA, USA). For each isolate, six wells were inoculated to ensure enough biofilm for subsequent RNA extraction. The inoculated plates were then incubated under microaerophilic conditions at 37 °C for 24 h.

#### 4.2.2. RNA Extraction

After incubation each well was washed with 100 μL of 1X phosphate-buffered saline (PBS) solution (Panreac, Barcelona, Spain) to eliminate planktonic cells, and the remaining biofilm was resuspended in 100 μL of 1X PBS by gently scraping the well bottom with a micropipette tip. Once resuspended, the content from each well was transferred into sterile 1.5 mL microtubes and centrifuged for 5 min at 5000× *g*. The supernatant was discarded and the resulting pellet was used for RNA extraction using the commercial RNeasy Mini Kit (Qiagen, Hilden, Germany), following the manufacturer’s protocol with slight modifications.

As part of the standard protocol, a DNase treatment was included using the RNase-Free DNase Set (27 U/sample) (Qiagen, Hilden, Germany), incubating the samples for 30 min at 25 °C to eliminate potential contaminating DNA residues.

Once the extraction was finished, the eluted RNA was further treated with TURBO™ DNase (4 U/sample) (Invitrogen, Carlsbad, CA, USA) to remove potential traces of genomic DNA. Samples were incubated for 30 min at 37 °C, following the manufacturer’s recommendations. After digestion, the enzyme was inactivated by adding 4 μL of 50 mM EDTA (Sigma, Ronkonkoma, NY, USA) and incubating at 75 °C for 10 min. The final RNA samples were aliquoted into appropriate volumes and stored at −80 °C until further use.

#### 4.2.3. Quantitative Real-Time Reverse Transcription PCR (RT-qPCR)

Gene expression quantification of the biofilm-related genes *luxS*, *fbps*, *otc*, *sadP*, *srtA* and *cpsE* was performed using the commercial SG OneStep qRT-PCR kit (EurX, Gdańsk, Poland) with SYBR Green as the fluorophore. The *16S rRNA* gene was used as a housekeeping gene. The primers used for each gene, as well as their respective functions, are summarized in Table S4 [37,50,51].

Reactions were carried out in 96-well plates (Applied Biosystems, Foster City, CA, USA), including technical triplicates for each gene and isolate. Gene expression was determined by the calculation of ΔCt (cycle threshold) values (target gene Ct—reference gene Ct), providing normalized relative expression levels for each gene. The thermal cycler used was the QuantStudio™1 Real-Time PCR System (Applied Biosystems, Foster City, CA, USA) and the cycling conditions are detailed in Table S5.

### 4.3. Antimicrobial Resistance Assays and Biofilm Characterization

#### 4.3.1. Preparation of Antibiotic Solutions

The selection of antibiotics was based on their relevance in veterinary medicine and their activity against *S. suis.* These antibiotics included ampicillin, clindamycin, ciprofloxacin, erythromycin and tetracycline (Sigma-Aldrich, Darmstadt, Germany). All antibiotic solutions were dissolved in water and prepared according to the manufacturer’s instructions.

Antibiotic concentration ranges were selected based on the expected activity range and the recommendations of the EUCAST guidelines [52], as summarized in Tables S1–S3.

ECOFFs (epidemiological cut-off values) for clindamycin, erythromycin and tetracycline were available for *S. suis*, while those for ampicillin and ciprofloxacin were inferred from *S. pneumoniae*. ECOFFs are epidemiological thresholds that separate wild-type (WT) isolates, those without phenotypic evidence of acquired resistance mechanisms, from non-wild-type (NWT) isolates, which may harbor such mechanisms. In this study, WT and NWT are therefore used in this epidemiological sense. Accordingly, NWT categorization should be interpreted as an approximation of acquired antimicrobial resistance based on MIC distributions and does not correspond to clinical breakpoints.

#### 4.3.2. Inoculum Preparation and Inoculation Using the Broth Microdilution Method

Bacterial biomass was resuspended in sterile saline solution (0.9% NaCl) and standardized to 0.5 McFarland (~1 × 10^8^ colony-forming units (CFUs)/mL). Then, 100 µL of this suspension was added to THB supplemented with 5% FBS. Two tubes were prepared per bacterial isolate and all assays were performed in duplicate.

For the broth microdilution method, 96-well polystyrene microfibre cell culture-treated plates (Corning Incorporated, Corning, NY, USA) were used to evaluate the inhibition of planktonic bacteria and biofilm formation assays. Antibiotic working solutions were serially diluted in supplemented THB to generate the desired concentration ranges. Then, 100 µL of the standardized bacterial suspension was added to each well. Plates were further incubated at 37 °C for 24 h under microaerophilic conditions. Bacterial growth was evaluated by visual turbidity and absorbance at 595 nm using a Multiskan^TM^ GO microplate spectrophotometer (Thermo Fisher Scientific, Waltham, MA, USA) and the minimum inhibitory concentration (MIC) was defined as the lowest concentration of antibiotics that inhibited visible growth. Each isolate–antibiotic combination was tested including two biological replicates; when the MIC values obtained in the two experiments were not identical, a third experiment was performed, and the final MIC was defined as the value observed in at least two experiments. The reference strain *Streptococcus pneumoniae* ATCC 49619 was included as a control.

#### 4.3.3. Biofilm Biomass Quantification Using the Crystal Violet Assay

Following incubation, biofilm biomass was quantified using the crystal violet (CV) staining method, based on an adapted protocol [28]. Briefly, the culture medium was carefully removed to preserve the biofilm. Next, 100 µL of a 2% CV solution (Montplet Esteban S.A, Barcelona, Spain) was added to each well and incubated at room temperature for 30 min on a microplate shaker (IKA, London, UK) to allow dye binding. Wells were washed three times with distilled water to remove excess dye and air-dried at 37 °C for 15 min. To solubilize the retained CV, 100 µL of 96.6% ethanol (Davila Villalobos, Valladolid, Spain) was added to each well. Absorbance was measured at 595 nm to quantify biofilm formation under different antibiotic concentrations.

CV data were normalized by calculating the mean and standard deviation (SD) of the negative control for each replicate, using the formula mean + 3 × SD. The resulting threshold value was subtracted from the absorbance readings. After normalization, wells with background-corrected absorbance ≤ 0 were considered negative for biofilm biomass, and the MIC for CV assay was defined as the lowest antibiotic concentration at which all biological replicates were negative; wells with higher corrected absorbance were considered positive and therefore did not meet MIC criteria. Each condition was evaluated in two biological replicates, and when the normalized CV values differed by more than 20% between them, the assay was repeated and a third experiment was performed; the mean of the available biological replicates (*n* = 2 or 3) was used for subsequent analyses.

#### 4.3.4. Cell Viability Assessment Using the MTT Assay

Biofilm cell viability after antibiotic exposure was assessed using the colorimetric MTT (3-(4,5-dimethylthiazol-2-yl)-2,5-diphenyltetrazolium bromide) assay [53]. To induce biofilm formation, 100 µL of the standardized bacterial suspension in THB supplemented with 5% FBS was added to each well of a 96-well plate, except for the negative controls, which received 200 µL of THB-FBS. Plates were incubated for 24 h at 37 °C under microaerophilic conditions. Following incubation, the culture medium was removed, the adherent cells were washed with 1X PBS and 200 µL of a freshly prepared antibiotic working solution (as previously described) was added. The plates were then incubated for another 24 h under the same conditions.

Wells were further washed with sterile 1X PBS to remove planktonic bacteria. Then, 100 µL of THB-FBS and 10 µL of 12 mM MTT solution (Fisher Scientific, Waltham, MA, USA) were added per well and incubated in the dark at 37 °C for 3 h. Afterwards, 85 µL of the supernatant was removed and replaced with 50 µL of dimethyl sulfoxide (DMSO) (Labbox, Barcelona, Spain) to dissolve the formazan crystals. Absorbance was measured at 550 nm to quantify the MTT formazan signal and at 620 nm as a reference wavelength for nonspecific background (plate, medium and residual turbidity); the 620 nm reading was subtracted from the 550 nm reading to obtain the net metabolic signal. Data normalization was carried out as described for the CV assay. The mean and SD of the negative controls were used to define a threshold as mean + 3 × SD, and this value was subtracted from the absorbance readings. After normalization, wells with background-corrected absorbance ≤ 0 were considered negative for MTT assay, and the MIC was defined as the lowest antibiotic concentration at which all biological replicates were negative; wells with higher corrected absorbance were considered positive and therefore did not meet MIC criteria. As for CV, each condition was tested in two biological replicates, and when normalized MTT values differed by more than 20% between experiments, a third experiment was performed and the mean of the available replicates (*n* = 2 or 3) was used in the analyses.

### 4.4. Statistical Analysis

All analyses were performed globally and by *S. suis* SV. For gene expression analysis, ΔCt values were converted to relative expression using the 2^(−ΔCt)^ formula and, subsequently, log_10_-transformed for normalization. Boxplots were used to represent gene expression levels across SVs. To assess whether significant differences in expression existed among SVs, the Kruskal–Wallis test was applied, followed by Dunn’s post hoc test when appropriate. Spearman’s rank correlation was used to evaluate associations between gene expression and biofilm formation levels in scatter plots, as well as to assess the significance of co-expression patterns in the correlation matrix. Biofilm formation levels quantified by CV staining were obtained from the data of a previous study conducted by our research group using the same isolates [28].

For antimicrobial susceptibility analyses, categorical WT/NWT data comparisons were carried out using Pearson’s chi-square test, with Fisher’s exact test applied when expected frequencies were too low. Post hoc pairwise comparisons were also performed with Fisher’s exact test. MIC values from the three methodologies were compared using the Friedman test, followed by Wilcoxon signed-rank tests when significant differences were observed.

All analysis and visualizations were performed using GraphPad Prism (version 10.5.0) [54]. Statistical significance was set at *p* < 0.05.

## Data Availability

Data is available on request from the corresponding author.

## Supplementary Materials

The following supporting information can be downloaded at: https://www.mdpi.com/article/10.3390/antibiotics14121224/s1, Table S1: Minimum inhibitory concentrations (MICs) of five antimicrobials against 60 *S. suis* isolates for the broth microdilution method (MD); Table S2: Minimum inhibitory concentrations (MICs) of five antimicrobials against 60 *S. suis* isolates for the crystal violet (CV) method; Table S3: Minimum inhibitory concentrations (MICs) of five antimicrobials against 60 *S. suis* isolates for the MTT method; Table S4: Primer sequences used for RT-qPCR analysis and corresponding gene functions [37,50,51]; Table S5: Thermal cycling conditions used in the RT-qPCR protocol.
