# Supplementary material for: Serovar-Dependent Gene Regulation and Antimicrobial Tolerance in Streptococcus suis Biofilms
Source: Antibiotics (Basel). 2025 Dec 4;14(12):1224. doi: 10.3390/antibiotics14121224 (PMC12729831; doi:10.3390/antibiotics14121224)
Supplement: Supplementary file 1 [file antibiotics-14-01224-s001.zip › antibiotics-3990048-supplementary.pdf]

Supplementary Materials

Table S1. Minimum inhibitory concentrations (MICs) of five antimicrobials against 60 *S. suis* isolates for the broth microdilution method (MD). The thick line represents the epidemiological cut-off value (ECOFF) used for each antimicrobial to classify isolates into non-wild type (NWT) and wild type (WT). Areas in grey represent values outside the concentrations of antimicrobial included in the broth microdilution method.

Broth microdilution (MD) method																				
Antimicrobial	Nº of isolates with MIC (µg/mL)																WT		NWT	
	0.008	0.016	0.03	0.06	0.12	0.25	0.5	1	2	4	8	16	32	64	128	256	n	%	n	%
Ampicillin	4	32	10	11	2	0	1	0									59	98.3	1	1.7
Ciprofloxacin							30	25	5	0	0	0	0	0	0		60	100.0	0	0.0
Clindamycin				11	1	0	0	0	0	0	3	45					12	20.0	48	80.0
Erythromycin			0	0	1	1	4	3	1	1	49						6	10.0	54	90.0
Tetracyclin								0	4	0	1	0	0	1	21	33	5	8.3	55	91.7

Table S2. Minimum inhibitory concentrations (MICs) of five antimicrobials against 60 *S. suis* isolates for the crystal violet (CV) method. The thick line represents the epidemiological cut-off value (ECOFF) used for each antimicrobial to classify isolates into non-wild type (NWT) and wild type (WT). Areas in grey represent values outside the concentrations of antimicrobial included in the broth microdilution method.

Cristal violet (CV) method

Antimicrobial	Nº of isolates with MIC (µg/mL)																WT		NWT	
	0.008	0.016	0.03	0.06	0.12	0.25	0.5	1	2	4	8	16	32	64	128	256	n	%	n	%
Ampicillin	6	20	14	13	5	0	0	1	1								58	96.7	2	3.3
Ciprofloxacin							32	19	4	2	1	0	1	1	0		58	96.7	2	3.3
Clindamycin				15	2	3	2	1	0	1	6	30					23	38.3	37	61.7
Erythromycin			6	6	1	0	5	5	2	4	31						18	30.0	42	70.0
Tetracyclin								2	6	0	2	0	2	5	16	27	10	16.7	50	83.3

Table S3. Minimum inhibitory concentrations (MICs) of five antimicrobials against 60 *S. suis* isolates for the MTT method. The thick line represents the epidemiological cut-off value (ECOFF) used for each antimicrobial to classify isolates into non-wild type (NWT) and wild type (WT). Areas in grey represent values outside the concentrations of antimicrobial included in the broth microdilution method.

MTT method																				
Antimicrobial	Nº of isolates with MIC (µg/mL)																WT		NWT	
	0.008	0.016	0.03	0.06	0.12	0.25	0.5	1	2	4	8	16	32	64	128	256	n	%	n	%
Ampicillin	1	15	6	6	3	3	0	7	19								31	51.7	29	48.3
Ciprofloxacin							8	9	7	2	1	2	0	5	26		27	45	33	55
Clindamycin				5	0	0	0	1	4	0	1	49					6	10.0	54	90.0
Erythromycin			0	0	1	0	3	1	1	4	50						5	8.3	55	91.7
Tetracyclin								1	0	1	1	0	1	3	11	42	3	5.0	57	95.0

Table S4. Primer sequences used for RT-qPCR analysis and corresponding gene functions.

Gene	Forward (5'-3')	Reverse (5'-3')	Gene function	Reference
16S rRNA	GTTGCGAACGGG TGAGTAA	TCTCAGGTCGGC TATGTATCG	Housekeeping gene. Encodes ribosomal RNA, a structural component of the small subunit essential for protein synthesis.	[50]
luxS	CGAGTTTGGAAG AAATTGCAG	AGCTGAATGAAG GCTGTGGT	Synthesizes autoinducer AI-2, regulating quorum sensing, biofilm formation and virulence.	[51]
fbps	AACCATCTTGCC AGGCTCCAC	CAGTTCAGAAGC CGTATCCCGAC	Encodes a fibronectin-binding protein that mediates adhesion to host tissues and colonization.	[50]
otc	TTGCCCTCTTGA AGCCATACCA	TTCCATTTCTTCT ACGCCGAAT	Produces ornithine carbamoylltransferase, involved in the arginine system and bacterial adhesion.	[37]
sadP	GGTGCACGTGAG ATTACCCT	TTTGGAGCTTCT GGCTCTGG	Encodes adhesin P, essential for binding to galactose residues during host colonization.	This study
srtA	TCGCTCATGTATG GAGCTGG	AACATCTGCCGC ACCAGTTA	Encodes sortase A, an enzyme that anchors surface proteins to bacterial cell walls.	This study
cps1E	AGTTGGACAGAA TGGACGCA	CTGGTTTTGGCT GAGCAAGTC	Produces a glycosyltransferase involved in capsule biosynthesis, varying among serovars and contributing to immune evasion. Each *cps* gene is specific to capsule synthesis of a given *S. suis* serovar.	This study
cps2E	AGTTGGACAGAA TGGACGCA	CATCCCACCTTG CATCTGGT		This study
cps7E	GGAGCGACTAAA CGTGTTGC	CATTCCCAAAAC GGACTGCC		This study
cps9E	GCGGCTAAAACT GCAAAGGT	GCAACACGTTTA GTCGCTCC		This study

Table S5. Thermal cycling conditions used in the RT-qPCR protocol.

Phase	Temperature	Time	Number of cycles
Reverse transcription	50 ºC	20 min	1
Initial denaturation	95 ºC	15 min	1
Denaturation	94 ºC	15 sec	40
Annealing and extension	60 ºC	60 sec	
Cooling	4 ºC	Indefinite	1
